# The potential of eugenol as a nematicidal agent against *Meloidogyne javanica* (Treub) Chitwood

**DOI:** 10.21307/jofnem-2020-103

**Published:** 2020-11-06

**Authors:** Eleni Nasiou, Ioannis O. Giannakou

**Affiliations:** Laboratory of Agricultural Zoology and Entomology, Department of Science of Crop Production, Agricultural University of Athens, Iera Odos 75, 11855, Athens, Greece

**Keywords:** Egg-differentiation, Egg mass*Meloidogyne* spp., , Sublethal doses activity, Terpenes

## Abstract

Root-knot nematodes (RKN; *Meloidogyne* spp.) are the most destructive plant parasites in vegetable production and their control is very challenging. This study aimed to define the nematicidal activity of eugenol on different life stages at 33.75 to 1,000 ppm doses against the root-knot nematode *Meloidogyne javanica* (Treub) Chitwood, 1949. This work is the first to report the effect of eugenol on egg differentiation and its vapor and sublethal doses activities. Second-stage juveniles (J2) were dead (99.5-100%) after 48 hr of exposure at a dose of 500 ppm. At this concentration, eugenol inhibited more than 70% nematode hatching. Additionally, the use of eugenol at sublethal doses reduced the number of females per gram in tomato roots in a pot test, and also inhibited egg differentiation. To the contrary, no nematostatic effects were observed in nematode motility bioassays. The phenolic monoterpenoid eugenol described herein merits further study as potential nematicide against the rootknot nematode *Meloidogyne javanica*.

Root-knot nematodes (RKN; *Meloidogyne* spp.) cause economic damage to a wide range of economically important open field and greenhouse vegetable crops and are considered one of the most damaging agricultural pests worldwide. The genus *Meloidogyne* has an extremely broad host range of over 2000 plant species, which can cause significant yield losses (Chitwood, 2003; Bleve-Zacheo et al., 2007).

The control of nematodes has become increasingly difficult due to many reasons. Many nematicides and soil fumigants have been removed from the market due to their toxicity and their harmful impact on the environment. An example is methyl bromide, a common fumigant that has been banned since 2005 (Karpouzas et al., 2007). The control against nematodes is further complicated by the lack of inherent resistant mechanism in most vegetable against nematode invasion or the cost-ineffective aspects of preventative approaches such as the long-term crop rotations or the scarcity of biological control agents in the market. As such, there is a great need for new alternatives (Sikora and Fernandez, 2005; Davies and Spiegel, 2011; Giannakou, 2011). In face of the recent European Union (EU) environmental restrictions, it is necessary to develop more ecologically rational alternative systems, like biological control and natural products (Faria et al., 2016).

In the actual context of research and development, a large number of essential oils and their constituents have been studied against a number of pests. In particular, terpenes, which are the primary volatile constituents of essential oils (EO), are among the most promising compounds for biorational pest control (Isman, 2000; Shaaya and Rafaeli, 2007; Sousa et al., 2015). One of these terpenes is eugenol (C_10_H_12_O_2_; 4-allyl-2-methoxyphenol) (Fig. 1), a volatile phenolic constituent and a flavoring agent of great economic importance in the food and cosmetic industries, mainly obtained from *Syzygium aromaticum* (commonly known as clove) (Chatterjee and Bhattacharjee, 2015).

**Figure 1:**
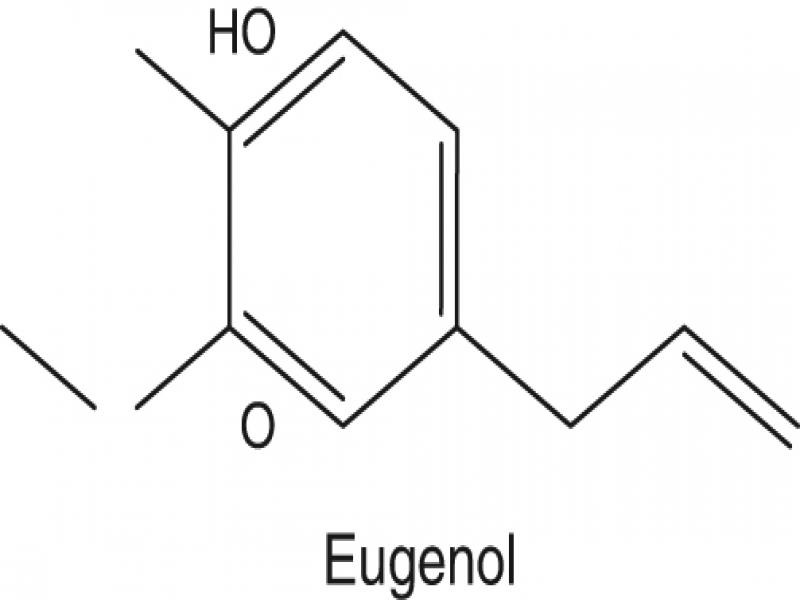
Molecular structure of eugenol.

Eugenol has shown interesting biological activities, such as antimicrobial (Marchese et al., 2017), anti-inflammatory (Daniel et al., 2009), antioxidant (Ogata et al., 2000), antibacterial (Da Silva et al., 2018), insecticidal (Park et al., 2000), and nematicidal (Tsao and Yu, 2000; Ntalli et al., 2010) among others. Previous lines of research have shown that eugenol at concentration of 660 µg/ml was nematotoxic to *Meloidogyne incognita* (Leela and Ramana, 2000). Ntalli et al. (2011) reported that mixtures of terpenes have significant synergistic effect against *Meloidogyne incognita* which cause paralysis. Nematostatic activity was evaluated to show efficacy of nematicides against root-knot nematodes (McGarvey et al., 1984).

The objectives of the present study were to determine (i) the nematicidal and nematostatic activity of eugenol on second-stage juveniles (J2) of *M. javanica*, (ii) the differentiation of eugenol inhibition on undeveloped eggs, (iii) the hatch inhibition activity of eugenol in egg masses, (iv) the effect of contact and vapor activity of eugenol on *M. javanica* infectivity, and (v) the effect of sublethal doses of eugenol on *M. javanica* infectivity.

## Materials and methods

### Nematode cultures

A population of *M. javanica* was reared on tomato (*Solanum lycopersicum* L.) cv. Belladonna in a greenhouse in Agricultural University of Athens, Greece and all seedlings were maintained in a greenhouse (25 ± 2°C, 16 hr photoperiod), in plastic pots (9 cm depth and 8 cm diameter) containing peat-based compost. The seedlings used for inoculations were 6 weeks old, at the four leaf stage. After 40 days, the inoculated plants were uprooted and the galled roots with mature egg masses were gently washed free of soil and cut into 2 cm pieces. Eggs of *M. javanica* were extracted with 1% sodium hypochlorite solution (Hussey and Barker, 1973). Second-stage juveniles (J2) were obtained by placing eggs on a Baermann funnel at the ambient temperature (27 ± 1°C). Freshly hatched J2 were obtained every 2 days and used in the experiments.

### Viability bioassays (nematicidal activity)

Solutions of eugenol were tested for J2 motility at the doses of 62.5, 125, 250, 500 and 1,000 ppm. Eugenol (Merck; Germany) was dissolved in ethanol (Sigma-Aldrich, Italy) and serially diluted in distilled water containing Tween-20 to produce test solutions of the above doses. In all cases, final terpene solutions were prepared containing double the test concentration. Eugenol solution (0.5 ml) was pipetted into each well of a Cellstar^®^ 24-well plate together with J2 suspension (0.5 ml containing approximately 40 J2) at a ratio of 1:1 (v/v), to produce the desired concentration of J2-terpene suspension. Tween-20 is a polysorbate nonionic surfactant and is used as an emulsifying agent for the preparation of stable oil-in-water emulsions. Final concentrations of ethanol and Tween-20 never exceeded 1 and 0.3%, respectively. Second-stage juveniles exposed to these concentrations of ethanol and Tween-20 were not affected, as preliminary tests and previous work indicate (Ntalli et al., 2010). As controls we used either distilled water or water with ethanol and Tween-20 at concentrations identical to those used in the treatment wells. All treated and control plates were covered with a lid to diminish terpene volatilization and incubated at 26 ± 1°C. Juveniles were observed with the aid of an inverted microscope (Zeiss, Germany) at 100 × magnification after 12, 24, 48, and 96 hr and were ranked into two distinct categories: motile or dead. For a window of 10 sec, we checked the juveniles for motility by probing them with a needle. Lack of movement was considered as a strong indication of juveniles’ death. The experiment was conducted twice and all treatments were replicated five times.

### Nematode motility bioassays (nematostatic activity)

Eugenol was dissolved in ethanol and diluted serially in distilled water containing Tween-20 resulting in solutions at the doses of 62.5, 125, 250, 500, and 1,000 ppm. In total, 50 ml of each test solution was placed in 250-ml Erlenmeyer flask and then newly hatched J2 were added. In all cases, working solutions were prepared containing double the test concentration and then mixed in flasks at a ratio of 1:1 (v/v) with a 50 ml suspension containing approximately 1,200 J2. A plastic tube connected to an air pump for oxygen supply was inserted into each flask. This was necessary to avoid lack of oxygen since each flask contained a high number of nematodes. Evaporation was avoided by covering the flasks with cotton plug and incubating at 26 ± 1°C temperature settings in dark. Solutions of water with ethanol (1%) and Tween-20 (0.3%) at the same concentrations as the ones in the treatment flasks as well as distilled water were used as controls.

After 12 hr, two solutions of 5 ml each were removed from every flask and used independently. The first one was divided into five aliquots of 1 ml each and placed into wells (containing approximately 40 J2 per ml/well). Second-stage juveniles were observed under an inverted microscope (100 ×) and ranked as motile or dead, as previously described. The second 5-ml solution was placed on a 38 μm sieve and J2 were rinsed with tap water to remove excess of eugenol. Then J2 were placed in a beaker and 5 aliquots of 1 ml each, containing approximately 35 J2, were placed in wells covered with a lid to avoid evaporation. Motile and dead J2 were counted under an inverted microscope (100 ×) after 12 hr to monitor recovery. The same procedure was repeated after 24, 48, and 96 hr. If any J2 regained motility, the effect was considered as nematostatic. Two solutions of 5 ml each was removed from flasks after 24, 48, and 96 hr and the same procedure, as described above, was followed. The experiment was conducted twice and all treatments were replicated five times.

### Effect of eugenol on egg development

Following the procedure described by Hussey and Barker (1973), we used sodium hypochlorite solution to extract *Meloidogyne javanica* eggs, from infested tomato roots (*Solanum lycopersicum* cv. Belladona). Eggs suspension was sieved through 53 and 38 μm, rinsed thoroughly with tap water and was collected into a 100 ml beaker. We estimated the number of eggs in the suspension using an inverted microscope (100 ×). Inoculum level was adjusted to 100 eggs per ml and used directly in the bioassay experiment.

The effect of eugenol solutions on the development of eggs at the doses of 62.5, 125, 250, 500, and 1,000 ppm was tested. Eugenol was initially dissolved in ethanol and brought to the desired volume using Tween-20 in water, as previously described. In all cases, working solutions were prepared containing double the test concentration and then mixed in Cellstar^®^ 24-well plates at a ratio of 1:1 (v/v) with suspension of eggs added to each well. Eggs suspension (0.5 ml containing approximately 50 eggs) was pipetted into each well and immediately eugenol solution (0.5 ml) was added. Distilled water and water with ethanol plus Tween-20 at concentrations equivalent to those in the treatment wells, served as the controls. All plates were covered with a lid to avoid evaporation and maintained at 26 ± 1°C. In total, 90% of eggs were undifferentiated at the beginning of the experiment. The number of either eggs having a fully developed juvenile or emerged J2 were counted in each well every 7 days (Tzortzakakis and Trudgill, 2005) using an inverted microscope (100 ×). For monitoring the egg development, eggs were observed on day 0 and were categorized either as differentiated (fully developed juvenile) or undifferentiated (eggs containing only cells). Undifferentiated eggs were considered those with cell division (one, two, or more cells).

The experiment was terminated after three weeks. It was conducted twice and each treatment was replicated four times.

### Effect of eugenol on egg hatching from egg masses

Mature egg masses were handpicked using sterilized forceps, from roots previously rinsed thoroughly, and placed in small plastic extraction trays made by 6 cm Petri dishes (one mature egg mass per extracting tray). Eugenol solutions (62.5, 125, 250, 500, and 1,000 ppm), initially dissolved in ethanol and brought to volume using Tween-20 in water (as described previously), were added to each extracting tray to cover the egg mass (10 ml eugenol solution/extracting tray). Egg masses were maintained for seven days and then test solutions were discarded. Then each egg mass was carefully submerged twice in clean water to remove excess of eugenol and finally was placed in extracting tray filled with clean water. The extracting trays were covered to avoid loss of water and placed in incubator at 26 ± 1°C. Hatched J2 were counted every week, they were discarded and the water was renewed with fresh one. The experiment was terminated after 5 weeks. Then every egg mass was removed from the extracting tray to a drop of water on a glass microscope slide, gently squashed with a coverslip and the number of unhatched eggs per egg mass was counted under an inverted microscope. The experiment was conducted twice and all treatments were replicated five times.

### Contact and vapor effect of eugenol against *M. javanica*

Sandy soil was collected from a field in Gargalianoi village, Messinia, Southern Greece. It was sieved using a 2 mm sieve to separate soil from debris and it was sterilized in an autoclave for 20 min at 100°C. Subsamples were removed to determine the soil moisture (oven drying at 50°C for 24 hr) and maximum water holding capacity (MWHC) (gravimetrical measurement following saturation of the soil with water and allowing to drain for 24 hr) (Pantelelis et al., 2006).

**Figure 2:**
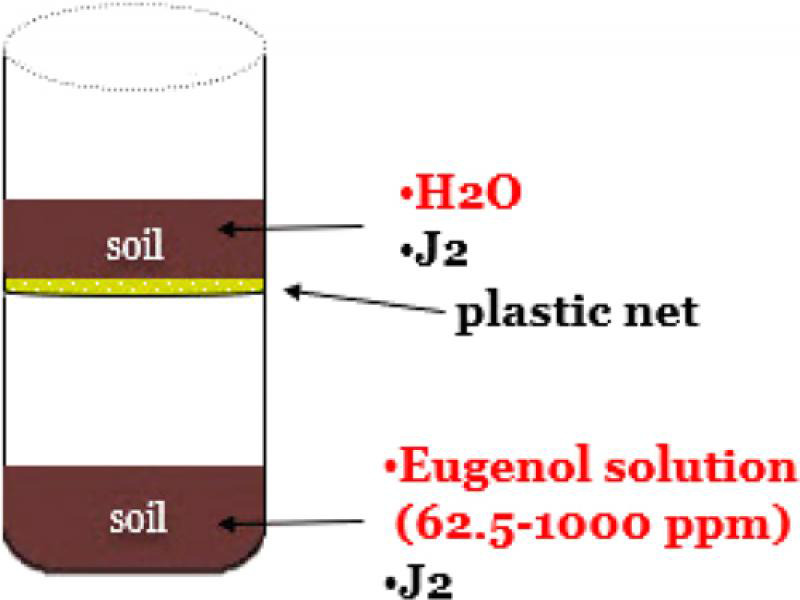
Pot trial methodology on the contact and vapor effect of eugenol against *M. javanica.*

In total, 48 plastic pots (7 cm depth and 5 cm diameter) were filled with 40 g soil each. Every pot was inoculated with a *M. javanica* suspension containing approximately 500 J2. In half of them, the bottom was replaced with a plastic mesh net (size 1 mm). Subsequently, eugenol solutions were added to the pots with plastic bottom (contact mortality bioassay), while plain water was added to the ones with the mesh net (vapor mortality bioassay). Each pot with a mesh net was placed on top of a pot with a plastic bottom, sealed with parafilm to prevent moisture loss and covered with aluminum foil to prevent light effect (contact-vapor mortality bioassay). The bottom of the netted pot was not in touch with the soil surface of the bottom pot so that nematodes could not migrate from the upper pot to the bottom pot (Fig. 2). The moisture content of the soil never exceeded the 20% of MWHC.

The efficacy of eugenol against nematodes was tested at concentrations of 62.5, 125, 250, 500, and 1,000 ppm. Eugenol stock solution was prepared in ethanol and Tween-20 (0.6%) to overcome insolubility, while distilled water with Tween-20 (0.6%) was used for further dilutions according to the above method. Control pots were consisted of soil with J2 only. The above experimental procedure was performed at two levels of temperature (20-22°C and 30°C).

For both temperatures, the plastic pots remained in climate room for three days. For the experiment at the 30°C, the plastic pots were placed on a metal tray with dimensions 60 cm × 40 cm × 8 cm (L × W × H). At the bottom of the metal tray a flexible silicone resistance was placed, connected with an automatic thermostat. Throughout the experiment the tray was filled with wet sand and plastic pots were immersed in the sand. After three days soil from every pot was removed and nematodes were extracted following a modification of Cobb’s decanting and sieving methods (Flegg, 1967) as suggested by Brown and Boag (1988). Juveniles were collected after 2 days and counted with the aid of an inverted microscope at 100 × magnification. The experiment was conducted twice and every treatment was replicated four times. Before statistical analysis, all numbers were calculated according to the Abbott (1925) formula:

$$\propto =\frac{\mathrm{mortality}\;\mathrm{treatment}-\mathrm{mortality}\;\mathrm{water}}{100-\mathrm{mortality}\;\mathrm{water}}\times 100\%.$$

### Sublethal doses effect of eugenol on juvenile invasion (pot experiment)

The efficacy of eugenol was evaluated using tomato seedlings cv. Belladona grown in plastic pots (10 cm depth and 6 cm diameter). All seedlings were 6 weeks old and at the four leaf stage. Initially, 15,000 newly hatched J2 were transferred from a graduate cylinder into four 250-ml Erlenmeyer flasks containing a total volume of 100 ml solutions of eugenol with concentration of 33.75, 67.5, 135, and 270 ppm and incubated at 26 ± 1°C. Eugenol was dissolved in ethanol (Sigma-Aldrich, Italy) and serially diluted in distilled water containing Tween-20 to produce test solutions of the above concentrations. Distilled water, as well as a solution of water with ethanol and Tween-20, at concentrations equivalent to those in the treatment flasks, served as control. After 24 hr, 1/3 of the suspension from each flask was placed in a 38 μm sieve and excess of eugenol was removed by washing with tap water. Then, nematodes were transferred to a beaker and four aliquots of 1 ml containing approximately 40 J2 were transferred to a 24-well plate. Second-stage juveniles were scored as motile or dead using an inverted microscope. The remaining J2 were maintained in the flasks for another 48 and 96 hr when the same procedure as previously described was repeated.

In total, 10 ml of a nematode suspension containing approximately 300 motile J2 was used to infect 6-weeks-old tomato plants. All plants were maintained for 30 days in a growth room at 26 ± 2°C. Plants were uprooted and roots were carefully washed free of soil and stained with acid fuchsin solution as described in Byrd et al. (1983). Roots were then washed in water and placed in vials containing equal volumes of glycerol and distilled water. The female nematodes were counted in the whole root system of each plant using a stereoscopic microscope at 12.5 × magnification. The experiment was conducted once in a randomized block design with five replicates per each treatment.

### Statistical analysis

All experiments were conducted using the completely randomized design. Data were subjected to one-way analysis of variance (ANOVA) using the General Linear Model (GLM). Treatments means were compared using the LSD test. Statistical analysis in all cases was conducted using SAS statistical package (SAS University Edition). All experiments (except the pot experiment) were conducted twice and they were combined and analyzed together since no variation was revealed between data.

## Results

### Viability bioassays (nematicidal activity)

The effect of eugenol on J2 motility of *M. javanica* is presented in Table 1. The percentage of dead J2 increased by increasing the exposure time. At the dose of 1,000 ppm, eugenol showed significant nematicidal activity; more than 99% of J2 were killed after exposure for 12 hr. Furthermore, eugenol gave more than 90% mortality at the 500 ppm dose after 24 hr, while at the same dose 99.8% of J2 were dead after 48 hr exposure. Additionally, approximately 80% of J2 were dead after 96 hr exposure at the dose of 250 ppm, while at doses of 125 and 62.5 ppm no significant effect on juvenile mortality was observed (Table 1). Dead juveniles had a specific shape defined as either straight (I-shape), bent (banana-shape), and L-shape which can separate between dead or motile nematodes.

**Table 1.** Effect of eugenol on the motility of *Meloidogyne javanica* J2 after immersion in test solutions at the doses of 1,000, 500, 250, 125, 62.5, and 0 ppm for 12, 24, 48, and 96 hr.

	Exposure time (hr)			
	12	24	48	96
Dose (ppm)	Dead J2 (%)	Dead J2 (%)	Dead J2 (%)	Dead J2 (%)
0	0 d	0.8 d	1.3 d	4.7 e
62.5	0.4 d	1.6 d	2.4 d	10.7 d
125	0.7 d	2 d	6.4 c	19.8 c
250	6.5 c	6.9 c	24.5 b	80.8 b
500	74.1 b	92.2 b	99.8 a	100 a
1000	99.5 a	100 a	100 a	100 a

**Notes:** Values are means of combined results from two experiments with five replicates each, since no significant differences were observed after ANOVA using the data of both experiments. Values followed by the same letter in a column do not differ significantly according to LSD (*P* < 0.001).

### Nematode motility bioassays (nematostatic activity)

No nematostatic effect was observed. The death of nematodes was further confirmed by maintaining all nematodes in wells with clean water (Cellstar^®^ 24-well plates) and evaluated after 12, 24, 48, and 96 hr. The percentage of dead J2 was similar to the previous experimental result when the nematicidal activity was tested in bioassay experiment (data not shown).

### Effect of eugenol on egg development

The inhibitory effect of eugenol in different doses on eggs differentiation after exposure for 21 days is presented in Table 2. The lower percentage indicates higher efficiency in inhibiting the egg differentiation. Eugenol at the doses of 500 and 1,000 ppm significantly inhibited eggs differentiation (66.2 and 21.4%, respectively) compared to control (92.1%). Also, there was no significant difference between the treatments of 62.5, 125, and 250 ppm compared to the control treatment (Table 2). The experiment was terminated since no further egg differentiation was observed in the control treatment.

**Table 2.** Effect of eugenol on the differentiation of *Meloidogyne javanica* eggs, after immersion of undifferentiated eggs at different doses of 1,000, 500, 250, 125, 62.5, and 0 ppm.

	Exposure time (21 days)
Dose (ppm)	Eggs differentiation (%)
0	92.1 a
62.5	90.6 a
125	90.5 a
250	82.1 a
500	66.2 b
1000	21.4 c

**Notes:** Values are means of combined results from two experiments with four replicates each, since no significant differences were observed after ANOVA using the data of both experiments. Values followed by the same letter in a column do not differ significantly according to LSD (*P* < 0.001).

### Effect of eugenol on egg hatching from egg masses

**Figure 3:**
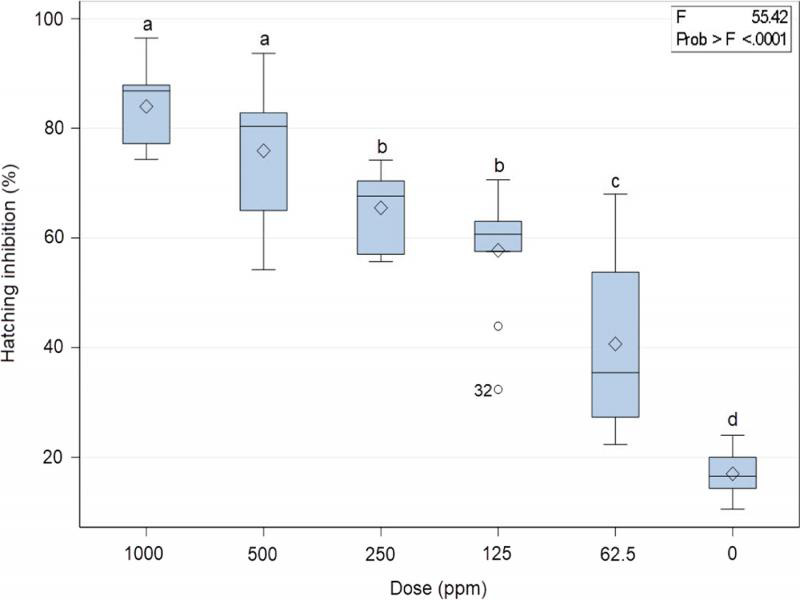
Effect of eugenol on *M. javanica* hatch, after immersion of egg masses at the dose rates of 1,000, 500, 250, 125, 62.5, and 0 ppm for 35 days. Values are means of combined results from two experiments with five replicates each, since no significant differences were observed after ANOVA using the data of both experiments. Samples in boxplots contain a large amount of variation among the treatment means relative to the amount of variation within the treatment. *F* statistic is defined as: (variation among the treatment means)/(variation among individuals in the same treatment). *F* value is large indicating that the means are significantly different and is evidence against the null hypothesis that assumes equal means. *F* is zero only when all group means are the same.

The highest number of hatched J2 (83%) was observed in egg masses remained in clean water (control) throughout the experiment (Fig. 3). Significantly fewer nematodes were hatched from egg masses treated with 500 and 1,000 ppm resulting in more than 70 and 80% fewer J2 counted, respectively, compared to the control. Eugenol also reduced at doses of 62.5, 125, and 250 ppm resulting in about 40, 57, and 65% hatching reduction as compared to the control treatment (Fig. 3). The experiment was terminated since not any more J2 were hatching.

### Contact and vapor effect of eugenol against *M. javanica*

**Figure 4:**
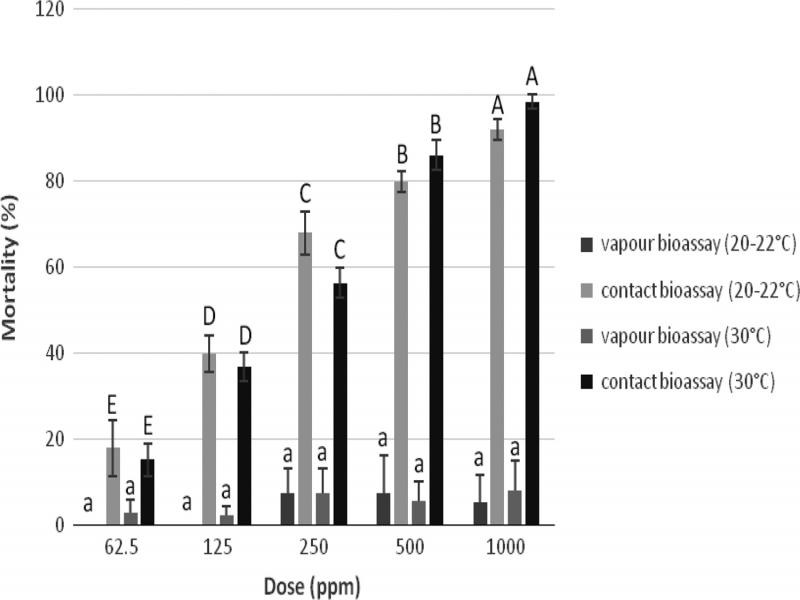
Effect of eugenol on contact and vapor mortality of *M. javanica* J2 in the soil, at the dose rates of 1,000, 500, 250, 125, and 62.5 ppm at 20 to 22°C and 30°C. Values are means of combined data from two experiments with four replicates each, since no significant differences were observed after ANOVA using the data of both experiments Error bars represent the standard deviation of mean. Bars with the same letter indicate no significant differences according to LSD test; upper case letters refer to the contact bioassay, lower case letters to the vapor bioassay.

The toxicity of eugenol on J2 was evaluated by the contact-vapor mortality bioassay (Fig. 4). As doses increased from 62.5 to 1,000 ppm there was a corresponding increase in J2 mortality. The number of J2 was significantly lower in contact bioassay at both levels of temperature. Eugenol at doses of 500 and 1,000 ppm showed strong contact mortality of J2 resulting in about 80 and 90%, respectively, in both levels of temperature. At a dose of 250 ppm, a 68 and 56% decrease of J2 in the soil was recorded in the contact bioassay at 20 and 30°C, respectively. On the other hand, a lower mortality was recorded at doses of 62.5 and 125 ppm in both temperature levels. In contrast, no vapor toxicity to *M. javanica* was observed, regardless the level of temperature.

### Sublethal doses effect of eugenol on juvenile invasion (pot experiment)

**Figure 5:**
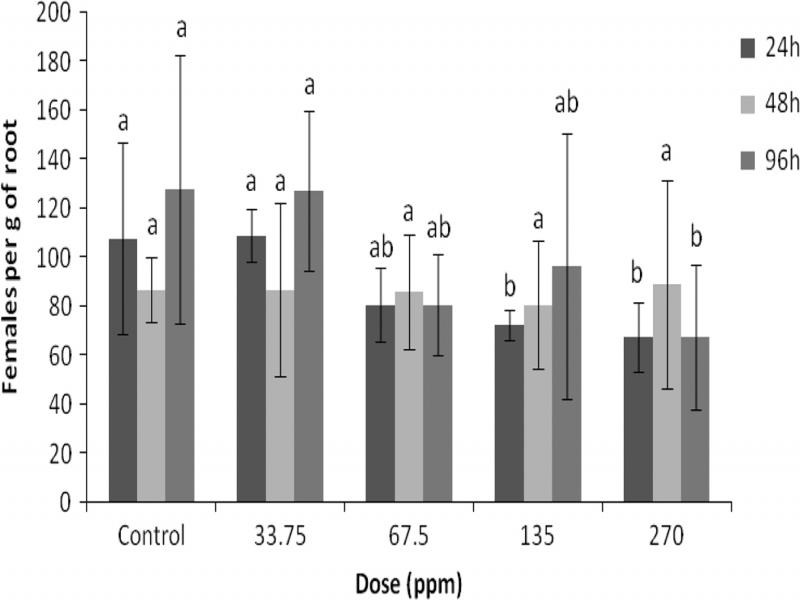
Numbers of females of *M. javanica* per gram of root after immersion in eugenol solutions at the dose rates of 0, 33.75, 67.5, 135, and 270 ppm for 24, 48, and 96 hr. Error bars represent the standard deviation of mean (*n* = 5). Bars having the same pattern with the same letter indicate no significant differences according to LSD test (*P* < 0.001).

The effect of sublethal doses activity of eugenol against *M. javanica* is shown in Figure 5. Eugenol showed potent sublethal dose activity at doses of 135 and 270 ppm, while at doses of 33.75 and 67.5 ppm there was no observed sublethal activity after exposure of 24 hr, compared to the control treatment (Fig. 5). Additionally, there were no statistical differences on female number in all doses of eugenol and control treatment after exposure for 48 hr (Fig. 5). However, the number of females decreased with increasing exposure time (96 hr). Particularly, at the dose of 270 ppm the number of females significantly decreased to 67, whereas at the control there were 127 females per gram of root, after exposure of 96 h (Fig. 5). Also, there were no statistical differences on female number between lower doses of eugenol (33.75, 67.5, and 135 ppm) and control after exposure for 96 hr.

## Discussion

Terpenes constitute the largest class of secondary metabolites in the plant kingdom (Dudareva et al., 2006) and they possess nematicidal activity against *Meloidogyne* and other important phytonematodes (Echeverrigaray et al., 2010; Nasiou and Giannakou, 2017, 2018). In this work, we investigated the nematicidal activity of eugenol found as main constituent of clove essential oil, against the root knot nematode *M. javanica.* Our results showed that the use of eugenol has a great potential in nematode control. The motility of *M. javanica* J2 was influenced by the time of exposure as well as concentrations of eugenol throughout the experiment. Our results confirmed the nematicidal properties exhibited by eugenol in vitro against the second-stage juveniles of *Meloidogyne javanica* (Treub) Chitwood and other plant parasitic nematodes (Sangwan et al., 1990). Ntalli et al. (2010) have previously reported 100% paralysis of *Meloidogyne incognita* J2 after exposure to eugenol at doses of 533 μL/mL for 24 hr.

This is the first report of eugenol’s inhibition on eggs differentiation using *Meloidogyne* species. Eugenol showed strong inhibition on egg differentiation; the majority of isolated eggs remained undifferentiated at a dose of 1,000 ppm after 21 days incubation and only 21.4% eggs were differentiated. The nematode eggshell consists of three layers; an inner lipid layer, a middle chitinous layer, and an outer vitelline layer (Bird and Bird, 1991). The eggshell is one of the most resistant biological structures known, which protects the eggs from attack by chemicals and biological nematicides (Wharton, 1980).

In more details, we observed a corresponding decrease in egg hatching of *M. javanica* with the increasing concentrations of eugenol, indicating dose-dependent activity. In the present in vitro studies, eugenol showed significant nematicidal efficacy against egg hatching of *M. javanica*. The maximum level of hatching inhibition was observed at 1,000 ppm (87.4% in experiment 1 and 80.6% in experiment 2) after 35 days of exposure. Carletti et al. (2011) reported that clove oil extract ABT-EU04^®^ (Xeda International S. A.), which has eugenol as its active ingredient and main constituent, even at the lowest concentration (0.125%) stopped the embryogenesis of unsegmented eggs. Only 0.96% of *M. incognita* J2 hatched from eggs in Test 1 and 0.69% in Test 2 while egg development stopped permanently after 4 days (Carletti et al., 2011). Egg hatch inhibition activity tested on egg masses is an indication of the terpene ability to penetrate the egg shell and thus inhibiting embryogenesis and further development.

We further tested the efficacy of eugenol which was found to exhibit strong contact mortality of *M. javanica* J2 in nematode infested soil. However, eugenol did not show significant fumigant nematicidal activity, compared to the control treatment. This is in agreement with previous studies (Nasiou and Giannakou, 2017, 2018) where carvacrol and geraniol have the same behavior in soil as eugenol.

The invasion of nematodes in roots was affected by treating J2 with sublethal doses. Most juveniles treated with eugenol remained motile, although some of them were not able to infect roots. Eugenol at doses of 270 and 135 ppm significantly reduced the number of females per gram of root compared to the control, after exposure of 24 and 96 hr (Fig. 5). Previous investigations have shown that eugenol at concentration of 1,500 mg/kg soil reduced the number of galls caused by *Meloidogyne arenaria* but had no significant effect on galls caused by *Meloidogyne incognita* (Walker and Melin, 1996).

The mechanism of action of EO and terpenes against nematodes remains unclear. Investigation on mode of action of EO and their constituents is important for nematode control, as useful information can be collected on the formulation and delivery means. According to Oka et al. (2000), there is a correlation between nematicidal and insecticidal activity and suggested the involvement of EO components in interrupting the nematode nervous system but also changing the permeability of the cell membrane.

In conclusion, phenolic monoterpenoid eugenol could be useful as a potential plant-based nematicide with contact action to control *M. javanica*. However, further study is needed to develop formulation, concentration and application times to improve eugenol efficacy under field conditions for practical use as novel nematicide or hatching inhibitor.
